# *In Vivo* Genotoxicity Assessment of Titanium Dioxide Nanoparticles by *Allium cepa* Root Tip Assay at High Exposure Concentrations

**DOI:** 10.1371/journal.pone.0087789

**Published:** 2014-02-04

**Authors:** Sunandan Pakrashi, Nitin Jain, Swayamprava Dalai, Jerobin Jayakumar, Prathna Thanjavur Chandrasekaran, Ashok M. Raichur, Natarajan Chandrasekaran, Amitava Mukherjee

**Affiliations:** 1 Centre for Nanobiotechnology, VIT University, Vellore, Tamilnadu, India; 2 Department of Materials Engineering, Indian Institute of Science, Bangalore, Karnataka, India; 3 Department of Chemical Technology, University of Johannesburg, Johannesburg, Gauteng, South Africa; RMIT University, Australia

## Abstract

The industrial production and commercial applications of titanium dioxide nanoparticles have increased considerably in recent times, which has increased the probability of environmental contamination with these agents and their adverse effects on living systems. This study was designed to assess the genotoxicity potential of TiO_2_ NPs at high exposure concentrations, its bio-uptake, and the oxidative stress it generated, a recognised cause of genotoxicity. *Allium cepa* root tips were treated with TiO_2_ NP dispersions at four different concentrations (12.5, 25, 50, 100 µg/mL). A dose dependant decrease in the mitotic index (69 to 21) and an increase in the number of distinctive chromosomal aberrations were observed. Optical, fluorescence and confocal laser scanning microscopy revealed chromosomal aberrations, including chromosomal breaks and sticky, multipolar, and laggard chromosomes, and micronucleus formation. The chromosomal aberrations and DNA damage were also validated by the comet assay. The bio-uptake of TiO_2_ in particulate form was the key cause of reactive oxygen species generation, which in turn was probably the cause of the DNA aberrations and genotoxicity observed in this study.

## Introduction

TiO_2_ nanoparticles are extensively used in manufactured products such as cosmetics, sunscreen, toothpaste and pharmaceuticals and deemed to have potential applications in electronics, optics, genomics, proteomics and bio-analytical fields because of their larger surface area, enhanced chemical reactivity and easier penetration potential into the cells [1]. TiO_2_ NPs can enter the aquifer from paint, sunscreen lotion, food additive, scrap plastic/glass/metalwares with a coating of nanoparticles [2]. The effluents from wastewater treatment plant were reported to be the main entry points of TiO_2_ NPs to the aquatic environment reported a direct evidence for the leaching of engineered TiO_2_ NPs into the surface water from paints [3]–[5]. National Institute for Occupational Health and Safety (NIOSH) has drafted a permissible exposure level (PEL) of 0.0015 µg/mL and a recommended exposure level (REL) of 0.0001 µg/mL for TiO_2_ NP based materials [6]. This necessitates the possible risks of TiO_2_ NP contamination to the surface water to be assessed [7].

Currently with increased concerns with environmental pollution, the *Allium cepa* test has emerged as a reliable tool for the prediction of environmental impact of disposed drugs, herbicides and also the engineered nanomaterials at the end of their life cycle. *Allium cepa* being sensitive and the wide availability, abundance, geographical distribution of the test species has made it a suitable candidate for exposure analysis. Several other test systems that fit the recommendations put down by the Committee on methods for toxicity tests with aquatic organisms, US EPA, can be utilized for exposure assessment. The *Allium cepa* test has been the most established plant assay system appraised by the US-EPA Gene-Tox Program for environmental monitoring of the toxicants [8]–[9].

The lipid peroxidation and generation of reactive oxygen species (ROS) causing DNA degradation in *A. cepa* root tip cells were reported by Kumari et al. (2011) using ZnO NPs [10]. A decreased mitotic index (60.3% to 27.62%) upon treatment with 100 µg/mL Ag NPs was also reported by the same authors [11]. The role of ROS (O_2_^•–^, H_2_O_2_) in DNA damage and cell death was further confirmed by Panda et al. (2011) using Ag NPs as toxicants [12]. In a recent study, Liman et al. (2013) reported the genotoxic effect of Bismuth (III) oxide nanoparticles (BONPs) on the *A. cepa* root cells [13].

The detailed genotoxicity studies involving exposure concentrations below 20 µg/mL of TiO_2_ NPs and the effect of internalized nanoparticles in *A. cepa* model is quite limited. Klancnik et al. (2011) proposed that the TiO_2_ NPs dispersed in distilled water may show higher toxicity towards *A. cepa* than in the growth media and the toxicity was duration and concentration dependent [14]. The impact of TiO_2_ NPs was found to have significant dependence on the medium of interaction. Nanoparticles in distilled water matrix exerted considerably higher toxic response compared to in defined growth medium. Micronuclei formation and chromosomal aberration due to TiO_2_ NP treatment was reported by Ghosh et al. (2010) at 320 µg/mL concentration, which was correlated with the decreased root size [15]. The researchers suggested lipid peroxidation as one the primary mechanisms involved in DNA damage upon NP treatment. However, none of these previous reports studied the relation between bio uptake of nanoparticles in the root tips and the ROS generation leading to consequent DNA damage.

The present study is the first of its kind to report on *Allium cepa* toxicity at high exposure levels of TiO_2_ NPs (12.5 µg/mL) with a linear dose dependence up to 100 µg/mL. The major focus of the study was to elucidate the possible mechanistic of genotoxicity. Based on the quantifiable data, the positive correlation between the internalization/bio-uptake of TiO_2_ NPs and oxidative stress with genotoxicity potential of the nanoparticles has been established. The detailed analyses by the optical microscopy, fluorescence microscopy and laser confocal microscopy, were employed to observe various aberrations like chromosome stickyness, chromosome breaks, laggard and clumped chromosome during various stages of cell cycle. The DNA damage owing to TiO_2_ NPs was confirmed through comet assay.

## Materials and Methods

### Chemicals

Dry Titanium (IV) oxide nano powder was obtained from Sigma Aldrich, USA. The physical characteristics of the particles according to manufacturer’s data are; size (<25 nm), purity: (99.7% anatase). Acetocarmine and Acridine Orange dyes were obtained from Himedia Labs, India. Dichlorofluorescin Diacetate (DCFH-DA) was procured from HiMedia Labs, India. All other reagents used in this study were of analytical reagent grade.

### Characterization of Nanoparticles

The TiO_2_ NPs were suspended in Millipore filtered water and dispersed by ultrasonic vibration (130 W, 20 kHz) for 30 min to produce four different concentrations at 12.5, 25, 50 and 100 µg/mL. These dispersions were subjected to dynamic light scattering analysis using a particle size analyzer (90 Plus Particle Size Analyzer, Brookhaven Instruments Corp, USA) to ascertain the hydrodynamic diameter in the test system.

### Test System and Treatment

For a continuous supply of the roots, healthy onion bulbs of Agrifound Red cultivar onions weighing 20–25 g each were grown in an enclosed chamber under the dark conditions. A temperature of 28±2°C was maintained during the period and provided with renewed water supply every 24 h. When the roots reached 2 to 3 cm in length they were treated with different concentrations (12.5, 25, 50, 100 µg/mL) of TiO_2_ NP suspensions for 4 h. The exposure was carried out under ambient lighting using white fluorescent lights with an illumination intensity of 970 lux and stirred at 150 rpm. Three replicates were made for each concentration for statistical validation of the observations.

### Microscopic Analysis

Freshly grown root tips were excised and exposed to TiO_2_ NPs dispersions. The micro slides were prepared by acetocarmine squash technique [16], [10],[11]. The root tips were kept in 1 N HCl for about 6 min followed by staining with 1% acetocarmine. Staining was continued for about 5–6 min. The slides were analysed at 1000X magnification for cytological changes. The mitotic index was calculated as the number of dividing cells per 1000 observed cells [17]. The number of aberrant cells was noted per total number of cells scored at each concentration [18].

#### Optical microscopy

The onion root tips were interacted with the TiO_2_ NPs solution for 4 h, washed with distilled deionised water. This was incubated with 1 N HCl solution for 6 min and stained with acetocarmine for 5–6 min. After the incubation, the roots were cut 1–2 mm from the tip, washed thoroughly to remove excess/unbound stain, and a squash was prepared observed under an optical microscope (Axiostar, Zeiss, Germany). The slides were analysed at 1000X magnification for cytological changes. The mitotic index was calculated as the number of dividing cells per 1000 observed cells [17].

#### Fluorescence microscopy

The onion root tips were interacted with the TiO_2_ NPs solution for 4 h. About 2 Root tips were then taken out and incubated for 2 min in the presence of 1 N HCl at room temperature. Tips were stained with nuclear specific stain acridine orange (AO) for visualization of chromosomal aberrations. To the root tips treated with 1 N HCl, 20 µL of AO (15 µg/mL in PBS) was added and incubated for 15 min under dark conditions. The suspension was then centrifuged at 6000 rpm for 10 min at 0°C, and the supernatant was discarded to eliminate unbound dyes after 5 min of incubation. The root tips were resuspended with Millipore filtered water. This was repeated three times in order to ensure complete removal unbound dyes. The root tips were placed on to a glass slide and covered with cover slip. Care was taken to avoid formation of air bubbles while placing the cover slip. Finally, the cover slip was pressed firmly with the help of thumb to prepare a uniform squash of the root tips. Dark condition was maintained throughout to avoid photo bleaching of dyes. Fluorescence images were observed using the BP 450–490, LP 590 filter using a fluorescence microscope (DM-2500, Leica, Germany). The images were recorded with the attached camera component (Leica-DFC-29) and processed using Leica-Application Suite 3.8.

#### Confocal laser scanning microscopy

Samples for confocal laser scanning microscopy were prepared following the same steps as fluorescence microscopy. The onion root tips were interacted with the TiO_2_ NPs solution for 4 h. About 2 Root tips were then taken out and incubated for 2 min in the presence of 1 N HCl at room temperature. Tips were stained with nuclear specific stain acridine orange (AO) for visualization of chromosomal aberrations. To the root tips treated with 1 N HCl, about 20 µL of AO (15 µg/mL in PBS) was added and incubated for 15 min under dark conditions. The suspension was then centrifuged at 6000 rpm for 10 min at 0°C, and the supernatant was discarded to eliminate unbound dyes after 5 min incubation. The root tips were resuspended with Millipore filtered water. This was repeated three times in order to ensure complete removal unbound dyes. The root tips were placed on to a glass slide and covered with cover slip. Care was taken to avoid formation of air bubbles while placing the cover slip. Finally, the cover slip was pressed firmly with the help of thumb to prepare a uniform squash of the root tips. Dark condition was maintained throughout to avoid photo bleaching of dyes. The chromosomal aberrations were then observed using confocal laser scanning microscope (Zeiss Lsm 510 Meta) equipped with 3 lasers for scanning the sample (Argon Ion, Helium/Neon I, and the Helium Neon II). The images were captured with the attached camera component and processed using (LSM 5 Image Browser).

### Statistical Analysis

Different phases of mitosis were counted and chromosomal abnormalities were observed to calculate mitotic index, phase indices and total abnormality percentage at the different phases of the cell division. Based on the observations the following indices were calculated in order to quantify the effects of TiO_2_ NPs.
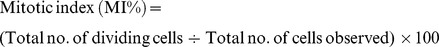


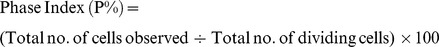



### Bio-uptake of TiO_2_ NPs

The treated *Allium cepa* root tips were washed with distilled deionised water and dried at 60°C for 24 h. After drying, the roots were powdered using sterile mortar and pestle. The powdered sample was digested using concentrated nitric acid and the soluble part was filtered through 0.45 µm membrane filter. The concentration of TiO_2_ NPs internalized into the roots was determined by Inductively Coupled Plasma-Optical Emission Spectroscopy (Perkin Elmer Optima 5300DV, USA).

Possible involvement of Ti^4+^ ions leached from nanoparticles was assessed. Dispersions of 12.5, 25, 50 and 100 µg/mL TiO_2_ NPs were incubated under test conditions for 4 h and nanoparticles were separated using a series of filtration through 0.1 µm membrane filter followed by 3 kDa filter to remove any suspended particles from the system. The filtrate was further analysed through particle size analyzer to ensure complete removal of particulate matter. The resultant suspension was subjected to ICP-OES analysis for detection and quantification of Ti^4+^ ions.

### Oxidative Stress Analysis

The disruption of cell wall due to generation of reactive oxygen species is one of the important mechanisms behind cell death [19]. After 4 h of treatment, 10 root tips were crushed in pre-chilled mortar and pestle using pre-chilled 1X PBS buffer. The solution was transferred to fresh falcon tubes and centrifuged at 10000 rpm for 10 min and the supernatant was transferred to a sterile 50 mL glass beaker and 5 µL (2′–7′-Dichlorofluorescin Diacetate (DCFH-DA) was added and kept in dark for 30 min. After 30 min the cell solution was analysed for ROS generation using fluorescence spectrophotometer (SL174, ELICO). The measurement was carried out at an excitation wavelength of 485 nm and emission wavelength of 530 nm.

### Comet Assay

The process was carried out following the modified protocol of Achary and Panda (2009) [20]. Alkaline comet assay was performed following an improved and simplified procedure that substituted phosphate-buffered saline buffer with Tris–HCl buffer. Analysis of comets was carried out employing fluorescence microscope (Leica DM-2500) and images were taken using the camera attached to the microscope (Leica-DFC-29). The comet images were visualized and captured at 100X magnification. Comets were analysed on the basis of olive tail moment of the comets using ‘casp comet tool’. The entire process of comet assay was carried out in dim or yellow light.

## Results and Discussion

### Characterization of TiO_2_ NPs Dispersion

The hydrodynamic diameter of the particles in the test system is of prime importance. The particles were found to have mean hydrodynamic diameters (MHD) of 92±3.6, 93.5±4.2, 92.8±4 and 94±3.1 nm at 0 h for 12.5, 25, 50 and 100 µg/mL concentrations respectively (Figure S1A, *Supplementary info*.). There were no significant differences between the MHDs of the dispersions of different NP concentrations at 0 h.

The hydrodynamic stability of the dispersions was evaluated for 4 h which corresponds to the duration of exposure to the root tips. The particles were found to have MHDs of 95.2±4.8, 96.8±2.5, 94.6±3.8 and 95.7±1.8 nm for dispersions of 12.5, 25, 50 and 100 µg/mL respectively (Figure S1B, *Supplementary info*.). The MHDs measured at 0 and 4 h did not have any statistically significant variation. Thus, the dispersions were found to be stable during the exposure period.

### Microscopic Analysis

#### Optical microscopic analysis

The optical microscopic analysis provided a detailed overview of the impact of TiO_2_ NP exposure. Upon exposure to varying concentrations of TiO_2_ NPs ranging from 12.5 to 100 µg/mL, the different features like chromosome stickyness, chromosome breaks, laggard and clumped chromosome were noted. About 5 replicates were analysed for each treatment group including untreated/control and 1000 cells were scored in each of the replicates. The frequency of occurances of different aberrant features upon exposure to increasing concentrations of nanoparticles and untreated group have been detailed in Table 1. The frequency of the anomalies showed a clear dose dependence where higher exposure concentrations caused higher number of anomalies. Interestingly a number of chromosomal aberrations and nuclear abnormalities were also observed at a concentration of 12.5 µg/mL. A strong dose dependent effect on mitotic index was noted. The mitotic indices were found to be 60.6±0.5, 52.4±0.4, 31.9±0.3 and 21.4±0.5% upon exposures to 12.5, 25, 50 and 100 µg/mL respectively. The untreated root tip cells showed a mitotic index of 69.0±0.4% (Table 2).

**Table 1:** Quantitative estimates of occurrences of different chromosomal aberrations observed in Allium cepa root tip cells after exposure to the different concentrations (12.5, 25, 50, 100 µg/mL) of TiO2 NPs dispersion and untreated/control.

Treatments	Chromosomal breaks (%)	Sticky chromosomes (%)	Laggard chromosome (%)	Clumped chromosome (%)	Chromosomal bridge (%)	Disturbed Anaphase/Metaphase (%)	Diagonal Anaphase (%)	Multipolar Anaphase (%)
Deionised distilled water (Control)	0±0	0±0	0±0	0±0	0±0	0±0	0±0	0±0
**12.5 µg/mL**	10.8±1.1	0±0	0±0	0±0	28.5±4.7	5.2±1.8	11.5±2	0±0
**25 µg/mL**	28.6±5.1	22.7±5.8	0±0	0±0	39.3±3.1	8.3±0.9	23.8±4.1	0±0
**50 µg/mL**	46.2±2.6	31.8±2.8	0±0	13.7±1.4	48.2±4.9	15.6±3.7	35.9±2.7	0±0
**100 µg/mL**	57.1±9.4	42.9±6.8	16.4±2.8	26.5±1.7	61.8±3.4	24.6±2.8	47.2±3.1	18.3±2.5

1000 cells were scored per treatment group. (n = 5).

**Table 2:** Mitotic Index in percentage (MI %) with the Standard Error (SE) for different exposure concentrations of TiO_2_ NPs where P, M, A, T stands for Prophase,Metaphase, Anaphase and Telophase as Phase Index in percentage respectively.

Treatments	Sample number (n = 5)	MI (%)	P (%)	M (%)	A (%)	T (%)	MEAN MI (%) ± SE
	Sample 1	69.5	61.2	2.9	2.1	3.3	
	Sample 2	70.3	62.0	2.5	2.3	3.5	
**Deionised distilled water (Control)**	Sample 3	68.1	60.5	2.0	2.9	2.7	69.0±0.44
	Sample 4	67.9	59.8	1.7	3.0	3.4	
	Sample 5	69.2	60.8	2.7	3.2	2.5	
	Sample 1	61.9	55.1	2.0	2.3	2.5	
	Sample 2	58.7	52.1	2.5	2.0	2.1	
**12.5 µg/mL**	Sample 3	61.3	54.2	2.9	2.4	1.8	60.6±0.54
	Sample 4	60.9	51.6	3.7	2.9	2.7	
	Sample 5	60.3	52.3	2.7	2.7	2.6	
	Sample 1	53.0	46.1	2.5	3.0	1.4	
	Sample 2	52.7	45.5	2.9	2.2	2.1	
**25 µg/mL**	Sample 3	51.8	44.9	2.8	2.0	2.1	52.4±0.46
	Sample 4	53.9	45.9	3.0	2.7	2.3	
	Sample 5	50.9	44.7	2.1	1.9	2.2	
	Sample 1	32.3	20.9	3.8	4.7	3.2	
	Sample 2	31.9	21.1	3.1	3.9	3.8	
**50 µg/mL**	Sample 3	30.7	22.5	2.9	2.5	2.8	31.9±0.31
	Sample 4	32.5	21.4	3.7	3.5	3.9	
	Sample 5	32.1	22.9	2.7	3.0	3.5	
	Sample 1	20.8	13.2	2.5	3.2	1.9	
	Sample 2	23.4	15.8	1.9	2.6	3.1	
**100 µg/mL**	Sample 3	21.7	13.9	2.2	2.7	2.9	21.4±0.53
	Sample 4	20.3	14.5	2.0	1.7	2.1	
	Sample 5	21.1	13.5	2.3	2.2	3.1	

**Figure 1:**
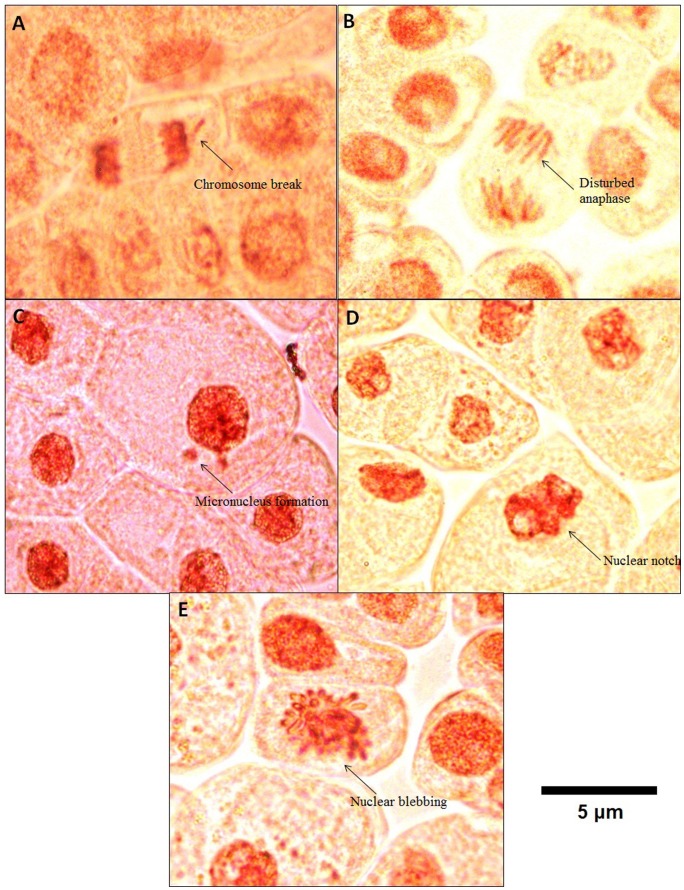
Various aberrant features observed upon exposure to 12.5 µg/mL (A) Chromosome break, (B) Disturbed anaphase, (C) Micronucleus formation, (D) Nuclear notch, (E) Nuclear blebbing.

Interestingly, an exposure to 12.5 µg/mL of TiO_2_ NPs showed occurance of sevral abnormal features. A chromosomal break was noted (Figure 1A) in addition to the other features such as disturbed anaphase (Figure 1B), micronucleus formation (Figure 1C) along with nuclear notch (Figure 1D) and nuclear blebbing (Figure 1E).

**Figure 2:**
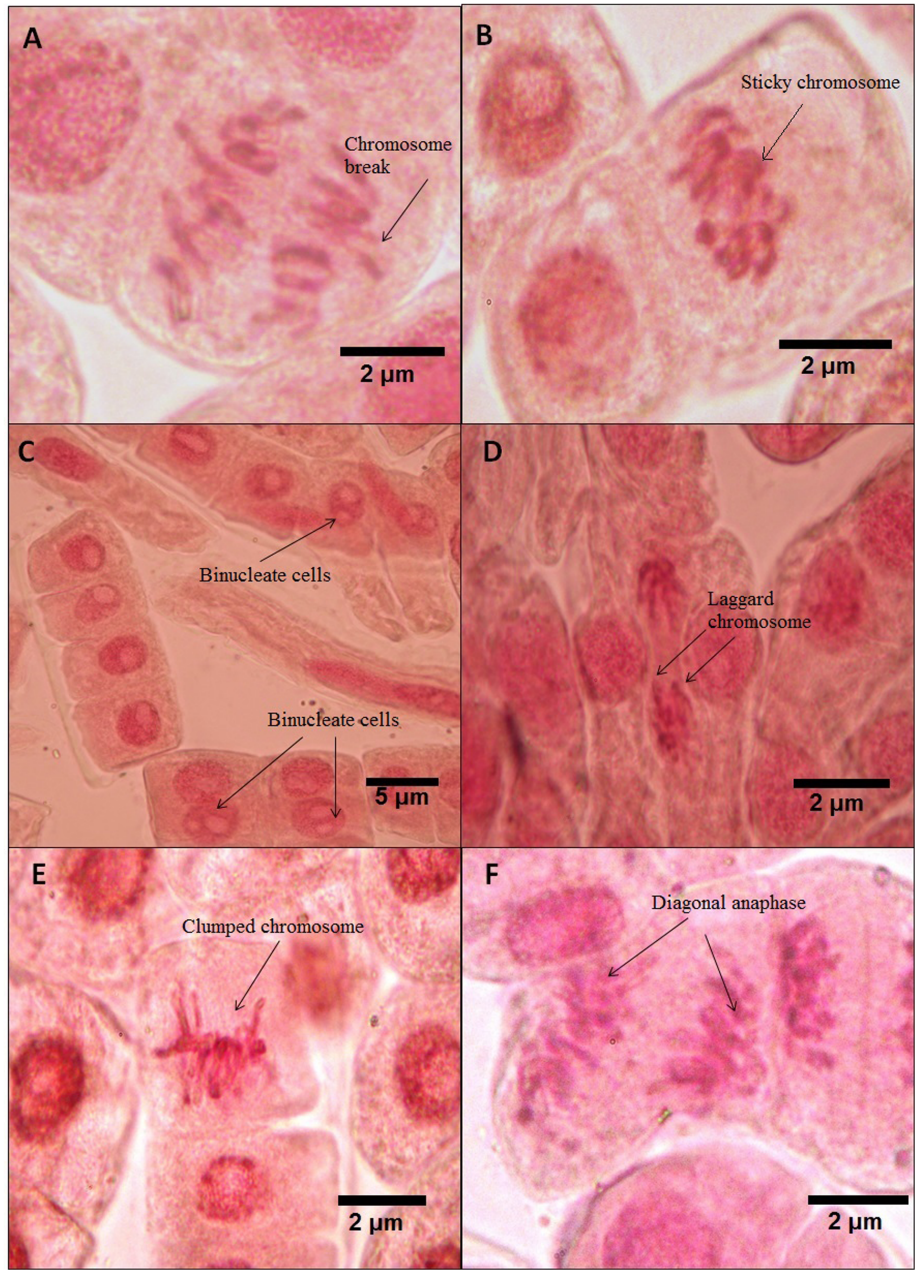
Various aberrant features observed upon exposure to 100 µg/mL(A) Chromosome break, (B) Sticky chromosome, (C) Binucleate cells, (D) Laggard chromosome, (E) Clumped chromosome, (F) Diagonal anaphase.

At 100 µg/mL exposure, several of the above mentioned anomalies were noted. Chromosomal break (Figure 2A), sticky metaphase (Figure 2B), formation of binucleated cells (Figure 2C), occurance of laggard chromosome (Figure 2D) and clumping of chromosome (Figure 2E), diagonal movement of chromosomes during anaphase due to improper polarization (Figure 2F) were visualised.

Similar features such as chromosome break (Figure S2A, *Supplementary info.*), sticky chromosomes during metaphase (Figure S2B, *Supplementary info.*), formation of binucleate cells (Figure S2C, *Supplementary info.*), clumping of chromosomes during metaphase (Figure S2D, *Supplementary info.*), diagonal anaphase (Figure S2E, *Supplementary info.*) upon exposure to 50 µg/mL TiO_2_ NPs. Additionally, formation of nuclear notch (Figure S2F, *Supplementary info.*).

Exposure to 25 µg/mL resulted in chromosomal break (Figure S3A, *Supplementary info.*), stickyness of chromosomes during metaphase (Figure S3B, *Supplementary info.*), chromosomal bridge formation (Figure S3C, *Supplementary info.*), diagonal anaphase (Figure S3D, *Supplementary info.*), disturbed anaphase (Figure S3E, *Supplementary info.*) and nuclear notch (Figure S3F, *Supplementary info.*).

#### Fluorescence microscopic analysis

**Figure 3:**
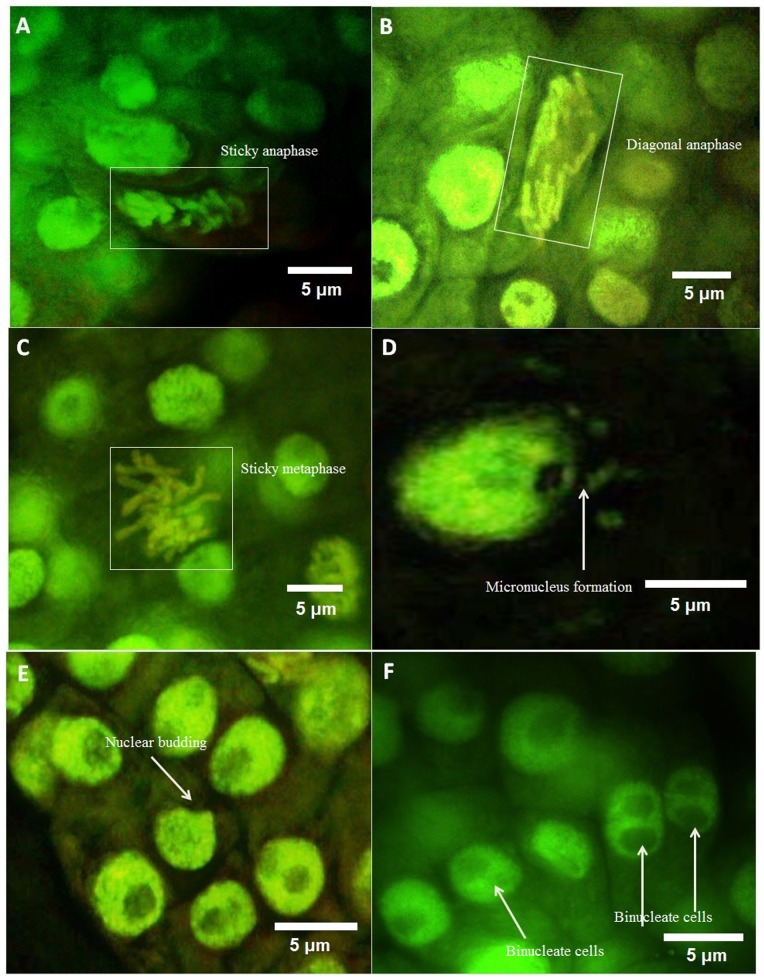
Various aberrant features observed under fluorescence microscope upon exposure to 12.5 µg/mL(A)Sticky anaphase, (B) Diagonal anaphase, (C) Sticky metaphase, (D) Micronucleus formation, (E) Nuclear budding, (F) Binucleate cells.

**Figure 4:**
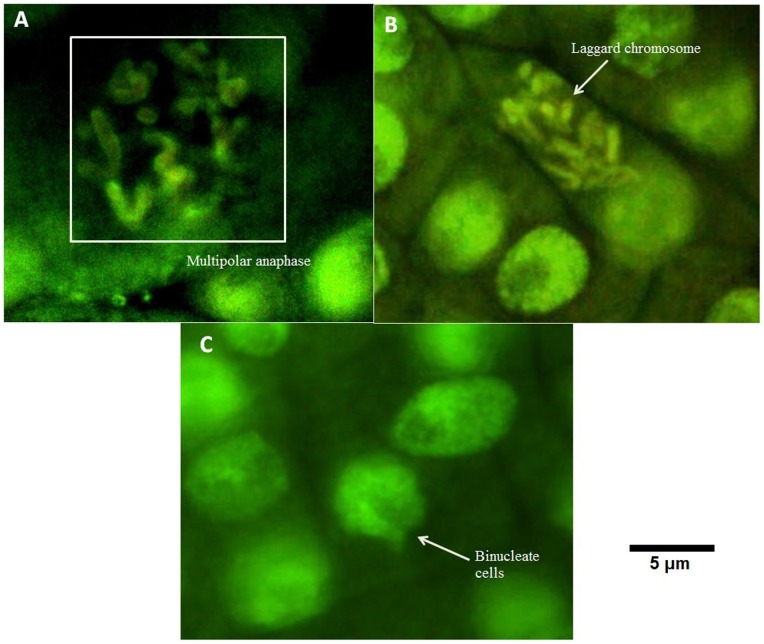
Various aberrant features observed under fluorescence microscope upon exposure to 100 µg/mL(A) Multipolar anaphase, (B) Laggard chromosome, (C)Binucleate cells.

At an exposure level of 12.5 µg/mL, the sticky anaphase (Figure 3A) and sticky metaphase (Figure 3B) were observed along with diagonal anaphase (Figure 3C). The nuclear abnormalities included the presence of more than one micronucleus in cells along with micronucleus during anaphase (Figure 3D). The occurrence of nuclear budding (Figure 3E) and bi-nucleate cells formation (Figure 3F) was also observed. At 100 µg/mL, the fluorescence microscopic images showed the chromosomal aberrations like multipolar (Figure 4A) and laggard chromosome (Figure 4B) at anaphase along with occurrence of bi-nucleate cell (Figure 4C).

#### Confocal laser scanning microscopic analysis

**Figure 5:**
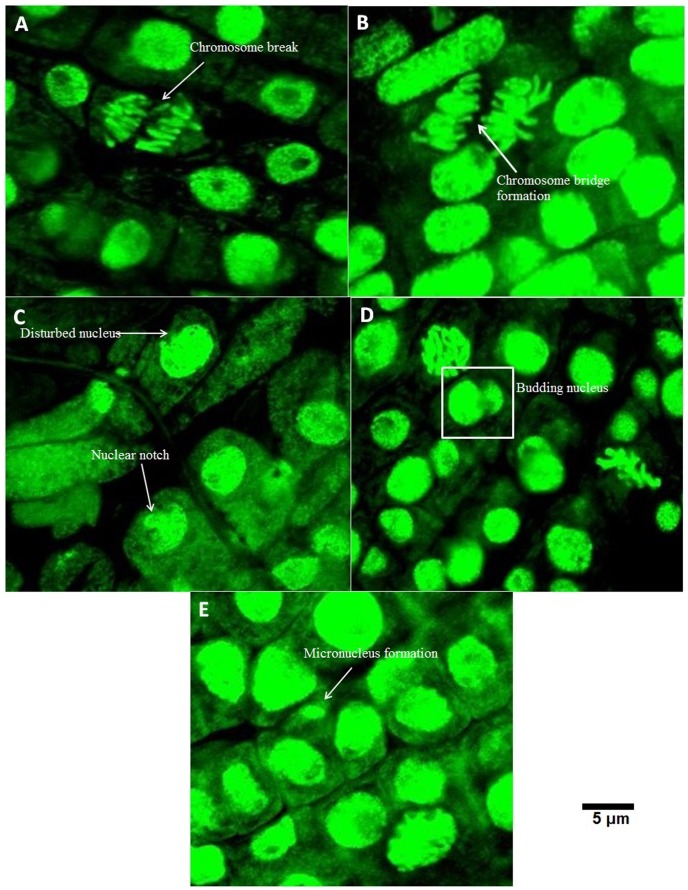
Various aberrant features observed under confocal laser scanning microscope upon exposure to 12.5 µg/mL(A) Chromosome break, (B) Chromosome bridge formation, (C) Disturbed nucleus and Nuclear notch, (D) Budding nucleus, (E) Micronucleus formation.

**Figure 6:**
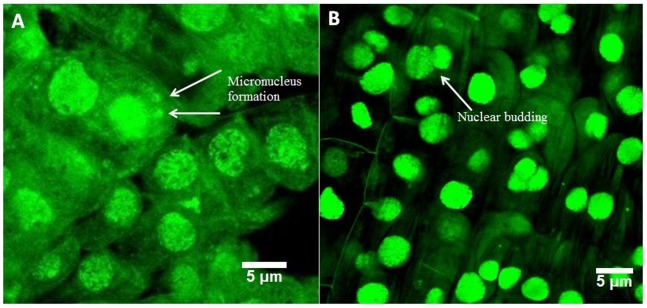
Various aberrant features observed under confocal laser scanning microscope upon exposure to 100 µg/mL(A) Micronucleus formation, (B) Nuclear budding.

The Confocal microscopic images provided a clear picture of the abnormalities observed in the optical and fluorescence microscopy. At 12.5 µg/mL concentration, chromosomal breaks were noted (Figure 5A). Formation of chromosomal bridges (Figure 5B) along with distorted (Figure 5C) and notched nucleus (Figure 5D) were noted. Furthermore, micronucleus formation (Figure 5E) and budding of nucleus (Figure 5F) were also observed.Exposure to100 µg/mL of TiO_2_ NPs showed a number of cells with micronuclei (Figure 6A) and certain cells with budding nucleus (Figure 6B). The observations were in close conjunction with other microscopic features elaborated in the previous sections.

The genotoxic effect of silver nanoparticles and role of oxidative stress induced cellular death have been demonstrated in recent past [12]. Silver nanoparticles have been found to cause genotoxicity on mammalian cell lines as well as in plant systems. The particles caused apoptosis and DNA break along with considerable elevation in oxidative stress level at 25 µg/mL exposure [20]. A recent report from our research group has shown the impact on mitotic index and subcellular organelles upon exposure to 25 µg/mL of ZnO nanoparticles [10]. Bi (III) nanoparticles have been proved to induce significant anomalies to the genetic material at a concentration of 12.5 µg/mL [13]. The occurrence of the aberrant features like chromosomal breaks, multipolarity, stickiness, laggard chromosome, bridge formation, micronucleus upon exposure to nanomaterials indicate their genotoxicity potential [20], [10].

Summing up the microscopic observations and comparing with previous findings, a significant genotoxic potential of TiO_2_ NPs was noted in the studied range of concentrations in the present work.

### Comet Assay

**Figure 7:**
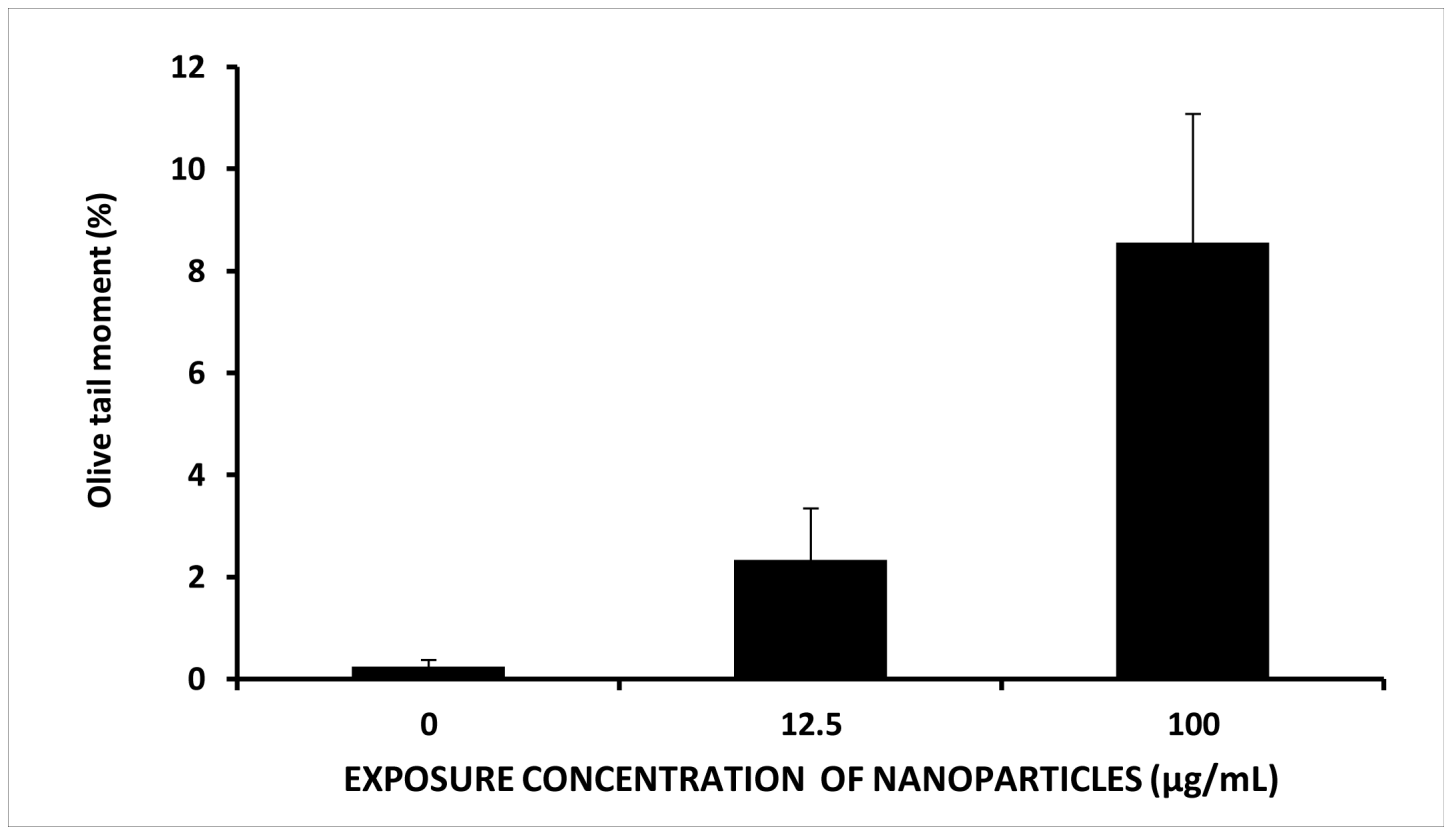
Comet data (% tail DNA) of *Allium cepa* treated with different concentration of TiO_2_ NPs.

The comet assay provided the extent of DNA damage which was quantified through olive tail moment. Olive tail moments of about 2.34±0.74 and 8.6±2.81% was observed at the test concentrations of 12.5 µg/mL and 100 µg/mL respectively indicating damaged DNA structure (Figure 7). The findings were in close correlation with the microscopic observations in the preceding sections demonstrating a wide range of distortions in the chromosomal orientations at the different phases of cell cycle. Chemical mediated genotoxicity due to oxidation of purine nucleotides is a major contributory factor towards DNA damage caused by TiO_2_ NPs [21]. To the best of our knowledge, the lowest reported exposure concentration of TiO_2_ NPs to exert a significant damage to DNA was 20 µg/mL [15].

### Bio-uptake of TiO_2_ NPs

**Figure 8:**
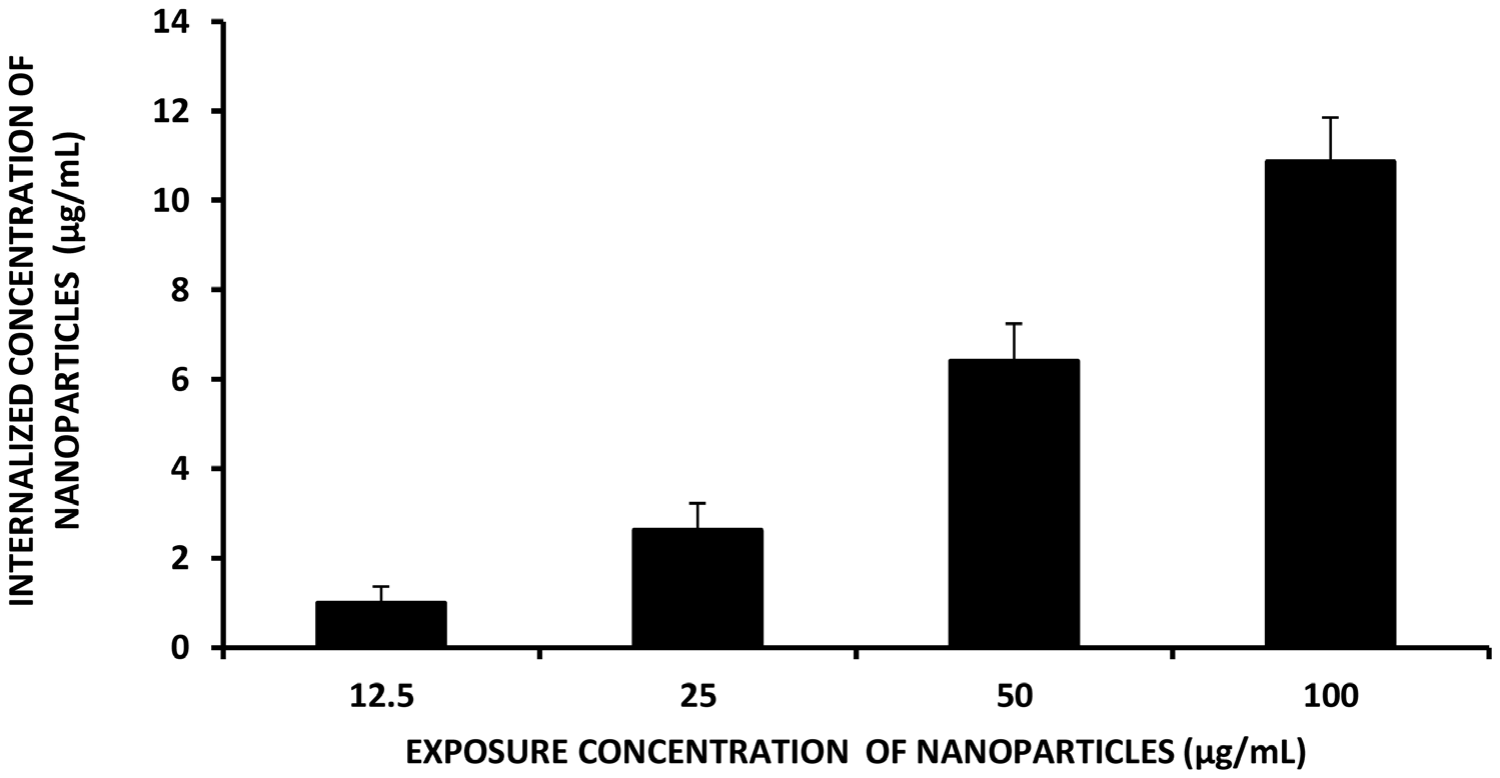
Internalization of TiO_2_ NPs into the root tips as quantified though ICP-OES.

The elemental analyses showed a dose dependant increase of TiO_2_ NPs internalized into the root cells of *Allium cepa.* We noted that1.67±0.3, 7.88±0.2, 10.48±0.1, 12.78±0.4 and 14.85±0.1 µg/mL of TiO_2_ NPs were internalized to the roots upon exposure to 12.5, 25, 50, 75 and 100 µg/mL concentrations respectively (Figure 8). The involvement of soluble Ti^4+^ ions was ruled out due to the insoluble nature of the TiO_2_ NPs which was substantiated by ionic analysis of the filtrate obtained after complete removal of suspended TiO_2_ NPs. Thus, the internalization of titanium was confirmed to be in particulate form and not in ionic state.

The internalization of the metal ions has been designated to be a prime causative of DNA damage leading cell death. The mechanism of damage has been attributed to elevated intracellular ROS levels [21]. Internalization of ZnO nanoparticles have been found to have detrimental effect on *A. cepa* leading to DNA damage [10]. However, the internalization of nanoparticles has been concluded in all these reports from the electron micrographs without a quantitative elemental analysis of internalized nanoparticles.

### Oxidative Stress Analysis

None of the prior reports on *Allium cepa* toxicity by the nanoparticles established any effective correlation between the extents of the internalized nanoparticles to elevation in ROS content resulting in anomalies in the genetic material.

**Figure 9:**
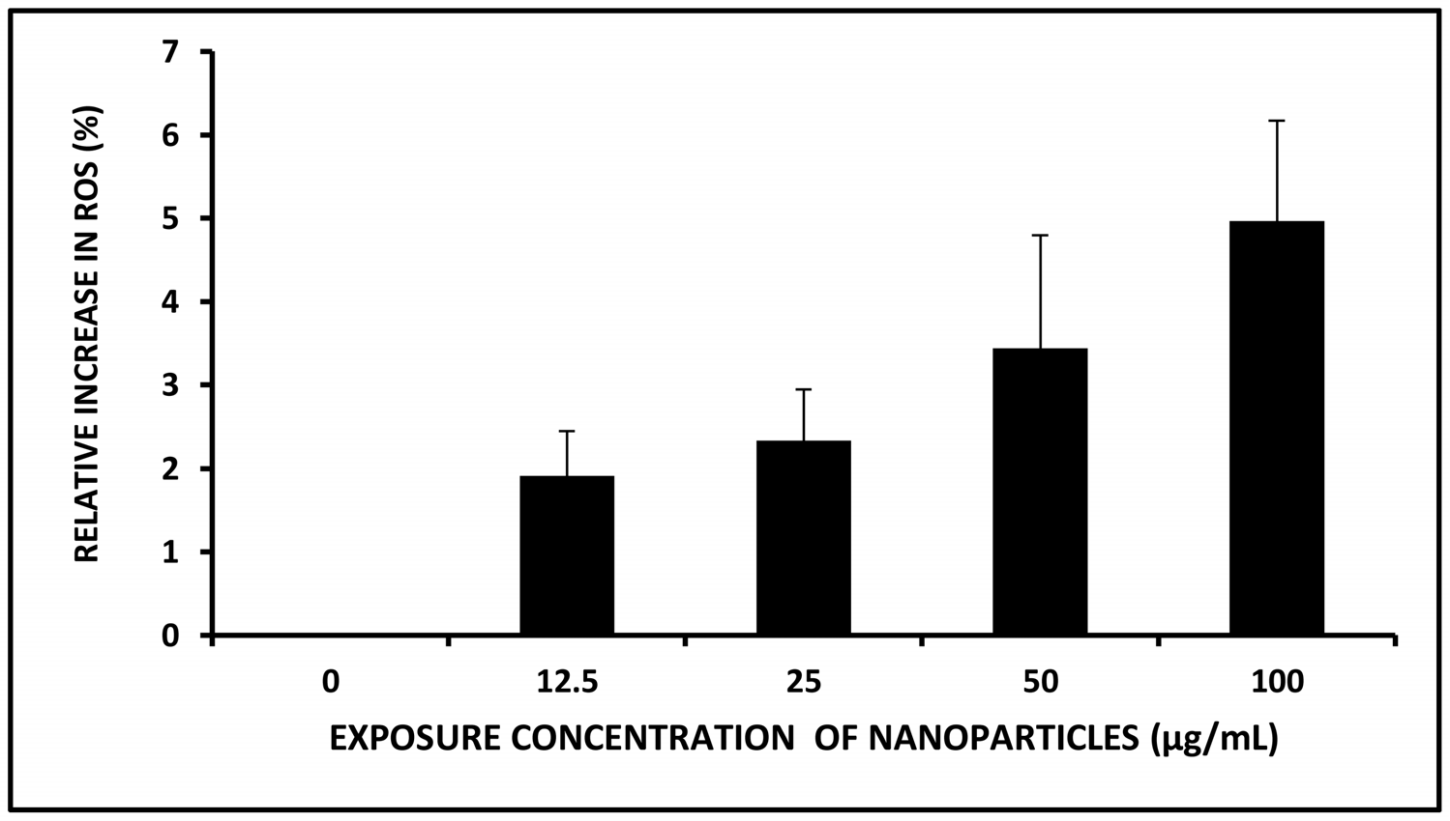
Oxidative stress profile upon exposure to TiO_2_ NPs.

The oxidative stress analysis by ROS assay also shows a concentration dependant increase in generation of intracellular reactive oxidative species. With respect to the untreated group, statistically significant increase in ROS content, i.e., 13.4±2, 22.1±1.2, 25.7±1.8 and 36.9±2.6% were noted upon exposure to 12.5, 25, 50 and 100 µg/mL TiO_2_ NPs respectively (Figure 9). This data correlated well with the dose dependent increase in the bio-uptake data as discussed in the preceding section. Therefore a clear conjunction between the nanoparticle uptake, increased generation of the ROS, and toxicity effects was established from this study. An imbalance in intracellular ROS content caused by nanoparticle exposure are well reported to induce DNA damages through oxidative stress owing to the oxidation of purine molecules [22]. Exposure to TiO_2_ NPs at 320 µg/mL dose has been reported to result in significant elevation of oxidative stress level inducing DNA damage in plant systems (*Allium cepa* and *Nicotiana tabacum*) as well as in human lymphocytes [15]. Oxidative stress leading to genotoxicity and cytotoxicity has also been reported by Phugare et al., (2011) [23]. Inhibition of various ROS scavenging enzymes and an elevated protein oxidation and lipid peroxidation profile indicated increase in oxidative stress level in *Phaseolus mungo* and *Sorghum vulgare* which were noted to be the major contributory factor towards genotoxicity and phytotoxicity [24]. All these reports suggested the role of oxidative stress in DNA damage. However, specific reports on the dose dependent relation of oxidative stress with DNA damage were absent.

## Conclusion

The observed chromosomal aberrations in conjunction with the comet assay results confirmed the dose dependent cytogenetic and genotoxic effects of the TiO_2_ NPs towards the *Allium cepa*. The dose dependent increases in the uptake/internalization of the particles and ROS have been established as major contributors towards the toxic effects. This study suggests that exposure to the TiO_2_ NPs are capable of inducing genotoxicity in the plant systems even at a low concentration of 12.5 µg/mL due to the internalization of the particles and the resultant oxidative stress.

## Supporting Information

Figure S1 **A:** Hydrodynamic size of TiO_2_ NPs at 0 h; **(B)** Hydrodynamic size of TiO_2_ NPs at 4 h.
(TIF)

Figure S2 Various aberrant features observed upon exposure to 50 µg/mL **(A)** Chromosome break, **(B)** sticky chromosome, **(C)** Bionucleate cells, **(D)** Clumped chromosome, **(E)** diagonal anaphase, **(F)** Nuclear notch, **(G)** Nuclear blebbing, **(H)** Nuclear degradation.
(TIF)

Figure S3 **Various aberrant features observed upon exposure to 25 µg/mL (A) Chromosome break, (B) sticky chromosome, (C) Chromosome bridge, (D) Diagonal anaphase, (E) Disturbed anaphase, (F) Nuclear notch.**
(TIF)
