# Correction: *In Vivo* Genotoxicity Assessment of Titanium Dioxide Nanoparticles by *Allium cepa* Root Tip Assay at High Exposure Concentrations

**DOI:** 10.1371/journal.pone.0098828

**Published:** 2014-05-20

**Authors:** The PLOS ONE Staff

There are errors in the Author Contributions. The correct contributions are: Conceived and designed the experiments: SP, AM. Performed the experiments: SP, SD, NJ, JJ, PTC. Analyzed the data: SP, AM, NC. Contributed reagents/materials/analysis tools: AM, AMR, NC. Wrote the paper: SD, SP, NJ, AM
