# Supplementary figures and images for: In Vivo Genotoxicity Assessment of Titanium Dioxide Nanoparticles by Allium cepa Root Tip Assay at High Exposure Concentrations
Source: PLoS One. 2014 Feb 4;9(2):e87789. doi: 10.1371/journal.pone.0087789 (PMC3913665; doi:10.1371/journal.pone.0087789)

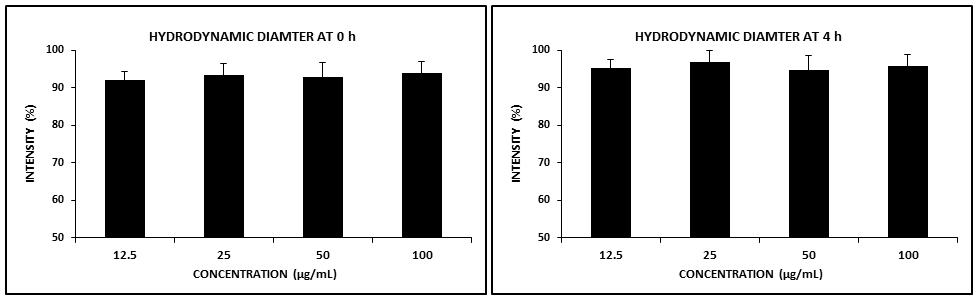

Supplement: Figure S1 — A: Hydrodynamic size of TiO2 NPs at 0 h; (B) Hydrodynamic size of TiO2 NPs at 4 h. (TIF) [file pone.0087789.s001.tif]

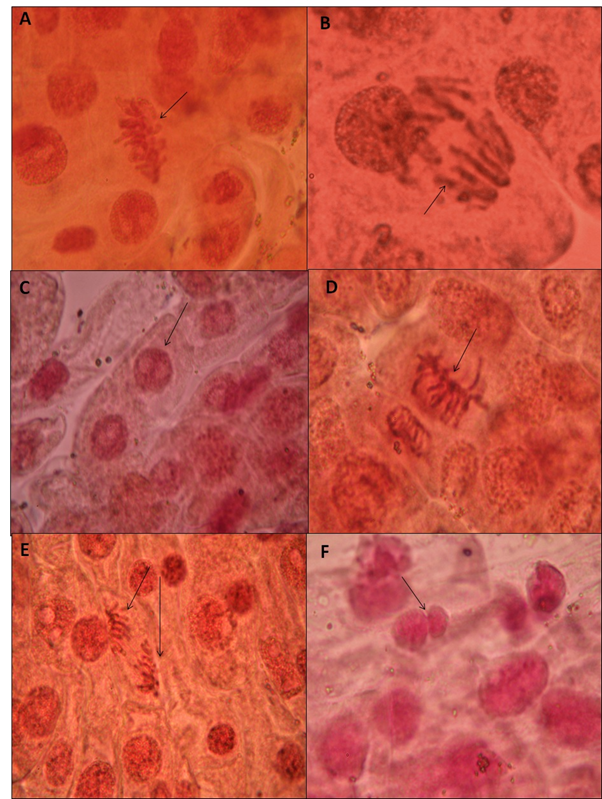

Supplement: Figure S2 — Various aberrant features observed upon exposure to 50 µg/mL (A) Chromosome break, (B) sticky chromosome, (C) Bionucleate cells, (D) Clumped chromosome, (E) diagonal anaphase, (F) Nuclear notch, (G) Nuclear blebbing, (H) Nuclear degradation. (TIF) [file pone.0087789.s002.tif]

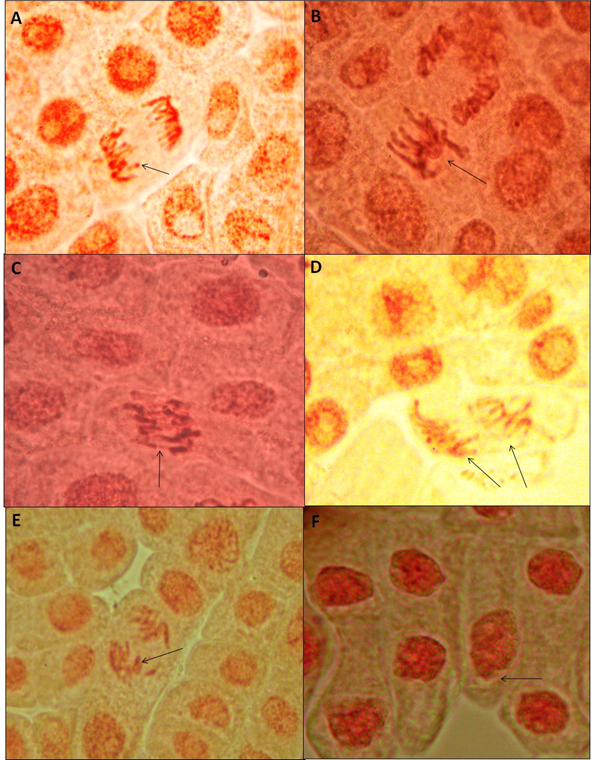

Supplement: Figure S3 — Various aberrant features observed upon exposure to 25 µg/mL (A) Chromosome break, (B) sticky chromosome, (C) Chromosome bridge, (D) Diagonal anaphase, (E) Disturbed anaphase, (F) Nuclear notch. (TIF) [file pone.0087789.s003.tif]
